# Job Satisfaction Among First-Generation Migrant Physicians in Anesthesiology and Intensive Care Medicine in Germany

**DOI:** 10.3390/healthcare12212107

**Published:** 2024-10-23

**Authors:** Mahmoud Elnahas, Jutta Hübner, Philip M. Lang, Emadaldin Ahmadi

**Affiliations:** 1 Department of Anesthesiology, Intensive Care Medicine, and Pain Therapy, Sozialstiftung Bamberg, 96049 Bamberg, Germany; philip.lang@sozialstiftung-bamberg.de; 2 Department of Hematology and Medical Oncology, Jena University Hospital, 07747 Jena, Germany; jutta.huebner@med.uni-jena.de (J.H.); emadaldin.ahmadi@med.uni-jena.de (E.A.)

**Keywords:** job satisfaction, first-generation migrant physicians, anesthesiology, intensive care medicine, germany, leadership communication, diversity strategies

## Abstract

Background/Objectives: This study examines job satisfaction, burnout, and well-being among first-generation migrant physicians in anesthesiology and intensive care medicine in Germany, comparing them to their native German counterparts. Methods: A cross-sectional survey design was utilized, collecting data from 513 physicians, 110 of whom identified as having a migration background. Job satisfaction was measured using the Warr-Cook-Wall (WCW) Job Satisfaction Scale, burnout was assessed with the Copenhagen Burnout Inventory (CBI), and well-being was evaluated using the WHO-5 Well-Being Index. Results: The job satisfaction ratings revealed no significant differences between migrant and German physicians in most dimensions, including physical workload, freedom to choose work methods, satisfaction with colleagues, responsibility, income, skill utilization, and variety in work tasks. However, migrant physicians reported significantly higher satisfaction with recognition received for their work and lower dissatisfaction with working hours. Burnout assessments showed that migrant physicians experienced higher psychological strain, perceiving every work hour as more exhausting and having significantly less energy for family and friends. Migrant physicians reported higher difficulty and frustration in working with patients. Well-being items indicated that migrant physicians felt less energetic and active but found their daily life more filled with interesting activities. Notably, the multivariate analyses of the total scale scores did not show significant associations between migration background and the overall outcome scales. Conclusions: The findings indicate unique challenges faced by migrant physicians, particularly in terms of recognition and patient-related burnout. These results highlight the need for targeted interventions to support migrant physicians, including cultural competence training and flexible working hours to enhance their job satisfaction and overall well-being. Addressing these issues is crucial for maintaining the quality of patient care and the occupational health of migrant physicians in Germany.

## 1. Introduction

The migration of physicians is a global phenomenon that has gained increasing attention over the years. In Germany, the number of foreign physicians has significantly increased, rising from 16,818 in 2007 to 59,893 in 2023 [1]. This influx has been crucial in filling vacancies, particularly in rural areas and in the eastern part of the country, where the shortage of medical professionals is more pronounced [2]. The State Chamber of Physicians in Saxony has highlighted the important contributions of foreign physicians in maintaining the quality of patient care and the operability of hospital wards, especially in underserved regions [3]. As such, immigrant physicians have become integral to the German healthcare system, particularly in hospital settings. While the professional satisfaction of physicians has been linked to patient satisfaction, there is a notable gap in the literature regarding the job satisfaction of immigrant physicians working in Germany.

Physicians’ health and well-being are critical quality indicators of healthcare systems, yet they are often neglected. Work stress can impair, while job satisfaction can elevate, the personal well-being of physicians. Factors contributing to physicians’ stress include high workloads, long working hours, and pressures that may be increased by exceptional situations such as the ongoing COVID-19 pandemic [4,5,6,7,8,9]. Studies have shown that stress, dissatisfaction, and burnout among physicians can lead to increased medical errors and a decline in the quality of care [10,11,12,13]. Moreover, job dissatisfaction can be a significant factor in physicians’ decisions to leave their positions or even to migrate to other countries where working conditions may be perceived as better [4,14]. In Germany, the healthcare system has undergone several reforms aimed at improving working conditions and reducing administrative burdens on physicians. Despite these efforts, challenges remain, particularly for immigrant physicians who may face additional hurdles such as cultural adaptation and language barriers [15]. The professional integration of migrant physicians is influenced by several factors, including the quality of leadership communication, the presence and effectiveness of diversity strategies, and the overall workplace culture [16,17]. Effective and inclusive communication from leadership is crucial for fostering a supportive work environment and enhancing job satisfaction among physicians. Additionally, well-implemented diversity strategies can help create an inclusive workplace where all employees feel valued and respected.

From the perspective of migration science, sociodemographic factors such as age, gender, educational background, and the duration of stay in Germany can significantly impact job satisfaction. The sense of inclusion and the ability to adapt to the cultural norms and expectations of the work environment are also critical factors. For first-generation migrant physicians, these aspects can pose significant challenges, affecting their overall job satisfaction and well-being. Physicians working in anesthesiology and intensive care medicine often face unique stressors due to the high-stakes nature of their work [18]. The need for precise and timely decision-making, coupled with the emotional toll of dealing with critically ill patients, can contribute to high levels of job stress and burnout [19]. For immigrant physicians, these challenges may be compounded by additional factors such as language barriers and differences in medical training and practices between their home countries and Germany.

Given these challenges, it is essential to explore how these physicians perceive their work environment and what factors contribute to their job satisfaction. By understanding these dynamics, healthcare institutions can develop targeted interventions to support their workforce better, ultimately leading to improved patient care and physician well-being. This study aims to fill this gap by exploring the job satisfaction, burnout, and well-being of first-generation migrant physicians in the fields of anesthesiology and intensive care medicine. By comparing these physicians with their native German counterparts, this research seeks to uncover the unique challenges and experiences faced by immigrant physicians and how these impact their job satisfaction.

To guide this investigation, we propose the following hypotheses:

**Hypothesis** **1:** *First-generation migrant physicians in anesthesiology and intensive care medicine experience significantly higher levels of psychological strain compared to their German counterparts.*

**Hypothesis** **2:** *The increased strain among migrant physicians has a significantly negative effect on their overall well-being and energy levels.*

**Hypothesis** **3:** *First-generation migrant physicians report significantly lower satisfaction with patient-related interactions and higher patient-related burnout compared to native German physicians.*

**Hypothesis** **4:** *First-generation migrant physicians perceive significantly less satisfaction with the recognition they receive for their work compared to their German counterparts.*

These hypotheses will guide the subsequent analysis, helping to frame the comparisons and provide deeper insights into the differences between migrant and native physicians. By testing these hypotheses, the study aims to provide evidence-based recommendations for interventions that can improve job satisfaction and well-being among first-generation migrant physicians in Germany.

## 2. Materials and Methods

### 2.1. Study Design

This study employs a quantitative research design to investigate the job satisfaction of first-generation migrant physicians in the fields of anesthesiology and intensive care medicine in Germany. The study population includes full-time employed migrant and German physicians to allow for a comparative analysis. A person was considered to have a migration background if they or at least one parent were not born with German citizenship. According to the definition used in the German Microcensus by the Federal Office for Migration and Refugees (Bundesamt für Migration und Flüchtlinge) since 2016, “A person has a migration background if they themselves or at least one parent did not acquire German citizenship by birth”. We followed this official definition of the German government.

Data were collected using a comprehensive questionnaire administered through SurveyMonkey, an online survey tool known for its robust features and user-friendly interface [20]. The link to the questionnaire was distributed via email to random samples of clinics across Germany that contain anesthesiology and/or intensive care departments. This method ensured a wide reach and accessibility, allowing for a diverse and representative sample of respondents. The survey link was sent to department heads and administrators in targeted clinics, who were asked to forward the invitation to their staff. Additionally, one reminder was sent after one month to encourage participation.

### 2.2. Questionaire

The survey included validated questionnaires to capture a broad range of factors influencing job satisfaction among first-generation migrant physicians. The questionnaire included items on sociodemographic characteristics, professional background, job satisfaction, perceived quality of leadership communication, and the effectiveness of diversity strategies in their workplaces. The questionnaire also included items on stress and burnout to provide a comprehensive understanding of the factors influencing job satisfaction. Additionally, items related to the sense of inclusion, cultural adaptation, and language proficiency were included to capture the unique experiences of migrant physicians. The questionnaire employed in this study is a comprehensive tool. The instrument is structured into multiple sections, each targeting specific domains relevant to the study’s objectives.

#### 2.2.1. Demographic Questions

This section collected basic demographic information about the respondents. It included questions regarding their migration background, such as whether they had a migration background, the country of their birth, and the number of years they had been living in Germany. Age and gender were also recorded to understand the demographic distribution of the respondents.

#### 2.2.2. Professional Background

The second section focused on the professional background of the respondents, including their qualifications, work experience, and current employment status. Respondents were asked about their highest medical qualification, the number of years they had been practicing medicine, and their current position (e.g., resident, specialist, consultant). The field of specialization (e.g., anesthesiology, intensive care medicine) was also queried.

#### 2.2.3. Tools and Their Characteristics

The questionnaire further employed various validated tools and scales to ensure the reliability and validity of the data collected.

Warr-Cook-Wall Job Satisfaction Scale [21]:

- Description: A German version of the Warr-Cook-Wall Job Satisfaction Scale, rated on a five-point Likert scale (ranging from “strongly disagree” to “strongly agree”). The overall job satisfaction calculated using the Warr-Cook-Wall (WCW) Job Satisfaction Scale is computed by averaging the scores across the 10 items.
- Usage: Widely used in research to measure job satisfaction.
- Validity: The scale has demonstrated good convergent and discriminant validity in several studies [21].
- Reliability: High internal consistency (Cronbach’s alpha typically > 0.80) and test-retest reliability [21].

Copenhagen Burnout Inventory (CBI) [22]:

- Description: Assesses personal, work-related, and client-related dimensions of burnout. The overall scale is computed by summing the individual items of the Copenhagen Burnout Inventory (CBI) and normalizing the total score to a 0–100 scale, where higher scores indicate greater levels of burnout.

Usage: Broadly applied in studies measuring burnout, particularly in healthcare settings [23,24,25].

- Validity: The CBI has strong construct validity and is recognized as a robust tool for measuring burnout [22].
- Reliability: High reliability with consistent Cronbach’s Alpha scores across studies (typically > 0.85) [22].

WHO Well-Being Index (WHO-5) [26]:

- Description: A short, self-reported measure assessing general well-being, consisting of five items rated on a six-point Likert scale (ranging from “at no time” to “all of the time”). The overall WHO-5 well-being score is computed by rescaling the individual item responses, resulting in a total score ranging from 0 (minimal well-being) to 25 (optimal well-being).
- Usage: Widely used internationally to measure psychological well-being.
- Validity: The WHO-5 has shown strong validity across diverse populations and settings [26].
- Reliability: High reliability with Cronbach’s alpha typically above 0.80 [26].

In addition to these standardized and validated tools, the questionnaire includes custom items developed to capture specific aspects of the work environment and the process of acculturation. These items were specifically designed as part of this study to address the unique aspects of acculturation among migrant physicians. The development process involved a thorough review of the existing literature on acculturation and language use, followed by expert consultations to ensure that the items were both relevant to the target population and comprehensive in capturing various dimensions of acculturation. The custom items related to acculturation aim to measure how well migrant physicians have adapted to the cultural norms and expectations of their workplace, including any cultural barriers they encounter. Additionally, the social integration questions were structured to assess social preferences and interactions, such as the origin of close friends, social visitors, and preferences in social gatherings. These items were designed to provide a detailed understanding of the social integration process among migrant physicians.

To ensure the reliability and internal consistency of the scales used in this study, we computed Cronbach’s alpha for each of the questionnaires applied to our sample.

- WHO Well-Being Index (WHO-5): The Cronbach’s alpha for the items in the WHO Well-Being Index was 0.865, indicating a high level of internal consistency and very good reliability for measuring general well-being among the participants.
- Warr-Cook-Wall (WCW) Job Satisfaction Scale: The Cronbach’s alpha for the items in the Warr-Cook-Wall Job Satisfaction Scale was 0.885, indicating excellent internal consistency and reliability in assessing job satisfaction within this sample.
- Copenhagen Burnout Inventory (CBI): The Cronbach’s alpha for the items in the Copenhagen Burnout Inventory was 0.893, demonstrating a high level of internal consistency and strong reliability in evaluating burnout across the personal, work-related, and patient-related dimensions.

These reliability coefficients confirm the robust psychometric properties of the tools used in this study and ensure that the measures are reliable for assessing the intended constructs in our sample. Exploratory factor analysis (EFA) was conducted on all items from the WHO-5 Well-Being Index, Copenhagen Burnout Inventory (CBI), and Warr-Cook-Wall (WCW) Job Satisfaction Scale using principal axis factoring with Varimax rotation. The Kaiser–Meyer–Olkin (KMO) measure of sampling adequacy was 0.950, indicating that the data were suitable for factor analysis. Bartlett’s test of sphericity was significant (χ^2^ = 10,104.847, *p* < 0.001), confirming that the correlations between items were adequate for performing factor analysis. Six factors were extracted, explaining a total of 63.898% of the variance. Items from the WCW Job Satisfaction Scale predominantly loaded onto Factor 1, while items from the CBI primarily loaded onto Factor 2. The WHO-5 items largely loaded onto Factors 3 and 4. These results suggest that the scales are measuring distinct but related constructs, supporting the validity of the scales in this sample.

### 2.3. Statistical Analyses

Job satisfaction was the primary dependent variable in this study. Independent variables included sociodemographic factors (e.g., age, gender, duration of stay in Germany), professional factors (e.g., speciality), and organizational factors (e.g., leadership communication, diversity strategies). All analyses were performed using SPSS version 29 (IBM Corp., New York, NY, USA). Continuous variables are presented as mean ± standard deviation, while categorical variables are shown as count and percentage. Normality was assessed using the Shapiro–Wilk test. Continuous variables were compared between groups using parametric or non-parametric tests, depending on the normality results. Categorical variables were compared using the chi-squared test. Pairwise comparisons for Likert-scale items were made using the Mann–Whitney U test. Bonferroni correction was used for multiple testing. Cronbach’s alpha was used to assess the internal consistency and reliability of the questionnaire items. Spearman’s rank-order correlation was performed to evaluate the strength and direction of associations between continuous and ordinal variables. Additionally, an exploratory factor analysis (EFA) was conducted using principal axis factoring with Varimax rotation to identify the underlying factor structure of the scales and ensure that the constructs measured were distinct. Univariate and multivariate linear regression models were employed to assess the relationships between potential confounders and the total scale scores for job satisfaction, burnout, and well-being. These regression models allowed us to account for potential confounding variables and better understand the independent contributions of each predictor. A *p*-value of <0.05 was considered statistically significant for all analyses.

## 3. Results

### 3.1. Demographic Characteristics and Professional Qualifications of Participants

The comparison between migrant and German participants revealed several significant differences (Table 1). Gender distribution differed significantly, with a higher percentage of males among migrants (60.0% vs. 52.9%) and a higher percentage of females among Germans (46.9% vs. 37.3%) (*p* = 0.012). Marital status also showed significant differences: a higher percentage of migrants were married (58.2% vs. 48.1%) (*p* = 0.006).

Religious affiliation was notably different (*p* < 0.001); migrants were more likely to be Muslim (30.0% vs. 1.2%) and less likely to be Christian (29.1% vs. 54.3%).

In terms of professional qualifications, significantly fewer migrants had emergency medicine qualifications compared to Germans (39.1% vs. 63.8%) (*p* < 0.001), and a higher percentage of migrants had no additional qualifications (50.9% vs. 31.0%) (*p* < 0.001). Migrants were also more likely to be specialist doctors (30.9% vs. 21.1%) and less likely to be assistant doctors (49.1% vs. 59.6%) (*p* = 0.048).

### 3.2. Descriptive Statistics Results for Migrant Participants

The questionnaires were submitted by the participants between 15 January 2024 and 22 May 2024. A total of 513 participants were included in the analyses. Out of the total 513 respondents, 110 (21.4%) reported having a migrant background, while 403 (78.6%) did not.

#### 3.2.1. Country of Birth

Most respondents were born outside of Germany; however, 11 respondents (10.0%) were born in Germany but identified as having a migrant background, indicating that they belonged to families with a migration history despite being born in Germany. The remaining respondents were born in various countries, including Egypt (12 respondents, 10.9%), Syria (6 respondents, 5.5%), Russia (8 respondents, 7.3%), Ukraine (4 respondents, 3.6%), and India (3 respondents, 2.7%). Other countries were represented by 1–3 respondents each.

#### 3.2.2. Duration of Residence in Germany

The average duration of residence in Germany among respondents was 12 years with a standard deviation of 11 years. For example, 6 respondents (1.2%) had been in Germany for 1 year, 7 respondents (1.4%) for 5 years, 12 respondents (2.3%) for 8 years, and 13 respondents (2.5%) for 10 years. Other durations ranged from 2 to 49 years, each represented by 1–4 respondents.

#### 3.2.3. Language Proficiency and Usage

The data show that a significant portion of respondents reported using both their native language and German equally across various contexts, including general language use (39.1%), thinking (30.0%), and when interacting with friends (27.3%). However, there was a notable tendency for language usage to be context-dependent. For instance, a majority of respondents (76.4%) reported using only their native language during childhood, while 40.0% listened exclusively to German radio programs (Table 2).

In terms of social integration, most respondents (52.7%) preferred social gatherings with a mix of people from both their home country and Germany. When it came to close friendships, 29.1% had an equal mix of friends from both backgrounds. Interestingly, a large majority (67.3%) expressed a preference for their children’s friends to come from both their home country and Germany, indicating a balanced approach to social integration among migrant physicians (Table 3).

### 3.3. Comparison of Migrant and German Participants

#### 3.3.1. Comparison of Job Satisfaction Ratings of Participants with and Without a Migration Background

The analysis of job satisfaction ratings between participants with and without a migration background revealed that most aspects of satisfaction did not differ significantly between the two groups (Table 4). However, two notable differences were observed: Participants with a migration background were significantly more satisfied with the recognition they received for their work (mean 3.17), whereas those without a migration background reported higher dissatisfaction (mean 3.75) (*p* < 0.001). Additionally, participants with a migration background expressed greater dissatisfaction with their working hours (mean 3.27) compared to those without (mean 3.62) (*p* = 0.035). Overall job satisfaction and other factors, such as physical workload, income, and the opportunity to use skills, showed no significant differences between the groups.

**Figure 1:**
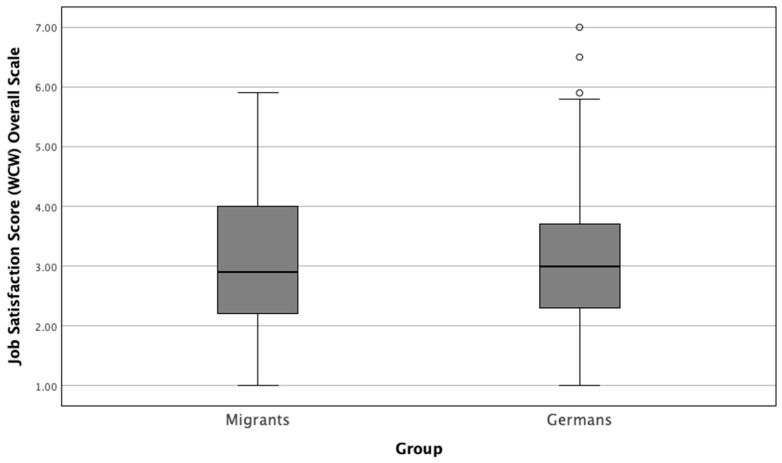
Comparison between overall job satisfaction scale scores of migrant and German physicians using the Warr-Cook-Wall (WCW) Job Satisfaction Scale (*p* = 0.725). This box plot illustrates the distribution of mean job satisfaction scores for migrant and German physicians, based on the Warr-Cook-Wall (WCW) Job Satisfaction Scale. Scores range from 1 (very satisfied) to 7 (very dissatisfied). The median scores are represented by the central line within each box, with the boxes indicating the interquartile range (IQR). Whiskers extend to the minimum and maximum scores within 1.5 times the IQR, and any outliers are represented by individual circles (“°”).

Next, we computed the overall job satisfaction scale scores using the Warr-Cook-Wall (WCW) Job Satisfaction Scale by averaging the scores across the 10 items. This approach provided a mean score ranging from 1 (very satisfied) to 7 (very dissatisfied), allowing for a direct comparison between the two groups. The mean WCW job satisfaction score for migrant physicians was 3.04 ± 1.16, while German physicians had a mean score of 3.07 ± 1.03 (Figure 1). A Mann–Whitney U test was conducted to compare the two groups, and the result was not statistically significant (*p* = 0.725), indicating that both migrant and German physicians reported similar levels of job satisfaction.

#### 3.3.2. Comparison of Burnout Ratings of Participants with and Without a Migration Background

The burnout ratings, assessed using the Copenhagen Burnout Inventory (CBI), revealed several significant differences between participants with and without a migration background. Both groups reported similar levels of tiredness, physical exhaustion, and emotional exhaustion, with no significant differences(Table 5). However, migrants were significantly more likely to feel they “can’t take it anymore” (*p* = 0.032), to perceive every work hour as exhausting (*p* < 0.001), and to have less energy for family and friends (*p* = 0.006). In terms of patient-related burnout, migrants reported greater difficulty working with patients (*p* = 0.001) and higher levels of being tired of working with patients (*p* < 0.001). They also wondered more often how much longer they could continue working with patients (*p* < 0.001). Overall, these findings indicate that migrants experienced higher levels of specific burnout symptoms, particularly related to work and patient interaction, compared to their German counterparts.

**Figure 2:**
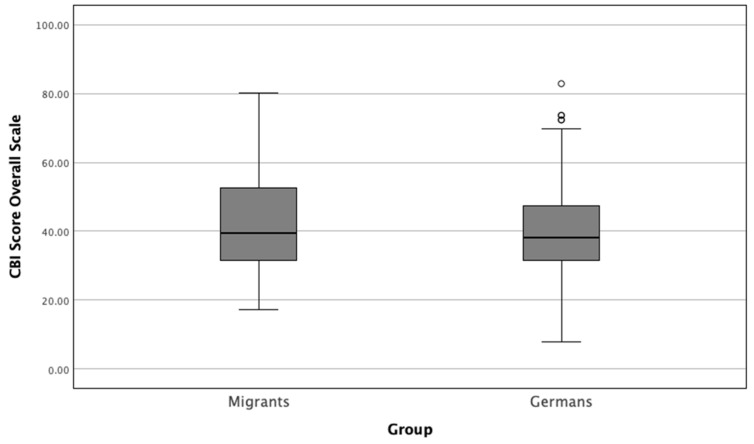
Comparison between Copenhagen Burnout Inventory (CBI) overall scale scores of migrant and German physicians (*p* = 0.219). This box plot illustrates the distribution of normalized burnout scores (CBI) for migrant and German physicians. The CBI scores have been normalized to range from 0 (no burnout) to 100 (maximum burnout). The median scores are represented by the central line within each box, while the boxes indicate the interquartile range (IQR). Whiskers extend to the minimum and maximum scores within 1.5 times the IQR, and any outliers are represented by individual circles (“°”).

Next, we computed the overall scale by summing the individual items of the Copenhagen Burnout Inventory (CBI) and normalizing the total score to a 0–100 scale, where higher scores indicate greater levels of burnout. This allowed for a more interpretable comparison between groups. The CBI scores were then compared between migrant and German physicians (Figure 2). Migrant physicians reported a mean burnout score of 41.98 ± 14.24, while German physicians had a mean score of 39.68 ± 12.96. A Mann–Whitney U test revealed that the difference between the two groups was not statistically significant (*p* = 0.219), indicating that both groups experienced similar levels of burnout as measured by the CBI.

#### 3.3.3. Comparison of Well-Being Ratings of Participants with and Without a Migration Background

The well-being ratings of participants with and without a migration background were assessed using the WHO Well-Being Index (WHO-5) (Table 6). Participants rated their experiences on a Likert scale where responses ranged from 1 (All the time) to 6 (At no time). Participants with a migration background had a mean rating of 2.80 ± 1.04 for being happy and in good spirits in the past two weeks, while those without a migration background had a mean rating of 2.94 ± 1.06. This difference was not statistically significant (*p* = 0.245). Regarding feeling calm and relaxed, participants with a migration background reported a mean rating of 3.19 ± 1.26, compared to 3.41 ± 1.16 for those without a migration background. This difference approached significance but was not statistically significant (*p* = 0.075). A significant difference was observed in feeling energetic and active. Participants with a migration background reported a mean rating of 3.24 ± 1.25, whereas those without a migration background reported a higher rating of 3.58 ± 1.18 (*p* = 0.008). For feeling fresh and rested when waking up, participants with a migration background had a mean rating of 3.72 ± 1.32, compared to 3.94 ± 1.32 for those without a migration background. This difference was not statistically significant (*p* = 0.097). A significant difference was found in ratings of having a daily life filled with things that interest them. Participants with a migration background reported a mean rating of 3.43 ± 1.24, while those without a migration background had a lower rating of 3.11 ± 1.21 (*p* = 0.017). In summary, the comparison of well-being ratings revealed significant differences in some aspects of well-being between participants with and without a migration background. Participants with a migration background reported lower levels of feeling energetic and active, but higher levels of having a daily life filled with interesting things. Other aspects of well-being, such as happiness, calmness, and feeling rested, did not show significant differences between the groups.

**Figure 3:**
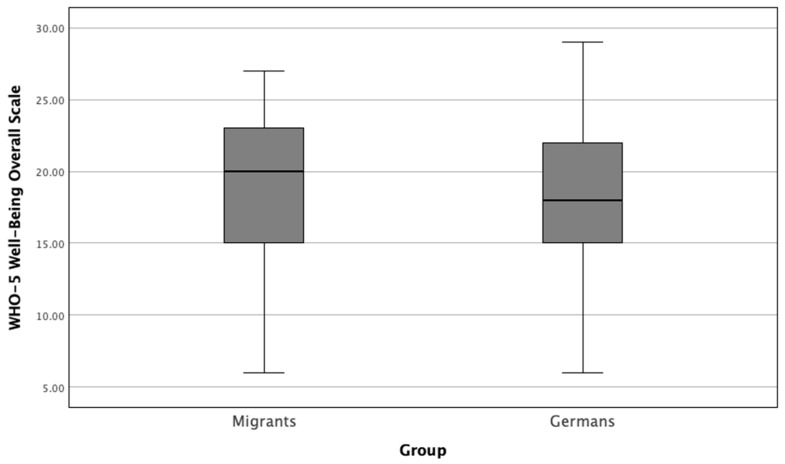
Comparison between WHO-5 well-being scores of migrant and German physicians (*p* = 0.154). This box plot illustrates the distribution of overall well-being scores (WHO-5) for migrant and German physicians. The WHO-5 scale ranges from 0 (minimal well-being) to 25 (optimal well-being). The median scores are represented by the central line within each box, while the boxes indicate the interquartile range (IQR). Whiskers extend to the minimum and maximum scores within 1.5 times the IQR. Outliers, if present, are represented by individual points.

Further, we computed the overall WHO-5 well-being scores by rescaling the individual item responses according to the literature, resulting in a total score ranging from 0 (minimal well-being) to 25 (optimal well-being). This rescaling was done to enhance the interpretability of the well-being scores across the two groups. The comparison of the WHO-5 between migrant and German physicians revealed no significant difference. The mean WHO-5 score for migrant physicians was 18.62 ± 5.07, while the mean score for German physicians was 18.01 ± 4.77. A Mann–Whitney U test was performed to compare the two groups, and the result was not statistically significant (*p* = 0.154). These findings suggest that the overall well-being of migrant and German physicians, as measured by the WHO-5 scale, was comparable (Figure 3).

### 3.4. Descriptive Statistics and Correlation Analyses of the Overall Scale Scores

Descriptive statistics for the aggregated WHO-5 well-being, Copenhagen Burnout Inventory (CBI), and Warr-Cook-Wall (WCW) Job Satisfaction scales are presented in Table 7. The mean WHO-5 well-being score was 18.14 ± 4.84, with a median score of 19.00, suggesting that participants generally reported moderate to high levels of well-being. The mean normalized CBI score was 40.17 ± 13.26, indicating moderate levels of burnout, with a median of 38.16. The mean job satisfaction score on the WCW scale was 3.06 ± 1.06, reflecting a moderate level of job satisfaction.

Correlation analyses, shown in Table 8, revealed significant relationships between the three scales. The WHO-5 well-being score was negatively correlated with both the normalized CBI score (Spearman’s Rho = −0.689, *p* < 0.001) and the WCW job satisfaction score (Spearman’s Rho = −0.559, *p* < 0.001), indicating that higher well-being was associated with lower burnout and higher job satisfaction. Additionally, a positive correlation was observed between the normalized CBI score and the WCW job satisfaction score (Spearman’s Rho = 0.586, *p* < 0.001), suggesting that greater burnout was associated with lower job satisfaction.

### 3.5. Multivariate Analyses

Univariate and multivariate linear regression analyses were conducted to investigate the relationship between various sociodemographic factors and the WHO-5 well-being overall scale score (Table 9). The pairwise analyses correlation analyses for dependent and independent variables are shown in Supplementary Tables S1 and S2. In the univariate analysis, significant associations were observed between the WHO-5 well-being score and several variables. Age was positively associated with well-being (ß = 0.056, *p* = 0.024). Female gender was associated with a lower WHO-5 score compared to male (reference: male) (ß = −1.424, *p* < 0.001). Additionally, living in a rural/small town (reference: urban) was associated with a lower well-being score (ß = −1.109, *p* = 0.011). Divorced participants reported lower well-being compared to married individuals (reference: married) (ß = −0.777, *p* < 0.001). Participants identifying as belonging to other religions (reference: none/agnostic/atheist) had significantly higher well-being scores (ß = 17.662, *p* < 0.001). The number of children was also positively associated with well-being (ß = 0.454, *p* = 0.012).

In the multivariate analysis, the effect of some variables remained significant while others lost significance when adjusted for other factors. Female gender continued to show a lower WHO-5 score compared to male, although the effect was not significant after adjustment (ß = −1.476, 95% CI = −2.353 to −0.599, *p* = 0.130). Living in a rural/small town (reference: urban) was marginally significant (ß = −0.872, 95% CI = −1.750 to 0.006, *p* = 0.052), and the impact of religious affiliation, specifically belonging to other religions (reference: none/agnostic/atheist), remained close to significance (ß = 3.388, 95% CI = −0.120 to 6.897, *p* = 0.058). Notably, the effect of age on well-being lost significance after adjustment (ß = 0.022, 95% CI = −0.039 to 0.082, *p* = 0.488).

In the univariate analysis of Copenhagen Burnout Inventory (CBI) Scores, several significant associations were identified (Table 10). Female gender was associated with significantly higher burnout scores compared to male (reference: male) (ß = 4.805, *p* < 0.001). Age was marginally associated with burnout, with younger individuals tending to report higher scores (ß = −0.123, *p* = 0.074). The number of children was inversely associated with burnout (ß = −1.339, *p* = 0.007), indicating that individuals with more children experienced less burnout.

In the multivariate analysis, after adjusting for other variables, female gender remained significantly associated with higher burnout scores (ß = 4.377, 95% CI = 2.002 to 6.752, *p* < 0.001). However, the association between age and burnout was no longer significant (ß = 0.006, 95% CI = −0.158 to 0.169, *p* = 0.947), and the effect of the number of children on burnout was attenuated and lost significance (ß = −0.988, 95% CI = −2.186 to 0.211, *p* = 0.106). Migration background showed a near-significant association with burnout in the multivariate model (ß = 2.601, 95% CI = −0.214 to 5.416, *p* = 0.070), suggesting that further investigation into this factor may be warranted. These findings suggest that female gender is a significant predictor of higher burnout, and having children may offer some protection against burnout, although this effect is diminished in the multivariate model. Age, on the other hand, does not appear to have a significant independent association with burnout after adjusting for other factors.

In the univariate analysis of Warr-Cook-Wall (WCW) Job Satisfaction Scores, age showed a marginal negative association with job satisfaction (ß = −0.008, *p* = 0.132), though this was not statistically significant (Table 11). Female gender was associated with higher job satisfaction scores compared to male (reference: male) (ß = 0.178, *p* = 0.058). Participants in a partnership (reference: married) reported higher job satisfaction compared to married individuals (ß = 0.225, *p* = 0.047), while chief doctors (reference: assistant doctors) were associated with lower job satisfaction scores (ß = −0.834, *p* = 0.019). Working in a university clinic (reference: public clinic) was positively associated with job satisfaction (ß = 0.303, *p* = 0.010).

In the multivariate analysis, after adjusting for other variables, the association between age and job satisfaction remained non-significant (ß = 0.006, 95% CI = −0.011 to 0.022, *p* = 0.498). Female gender continued to show a positive, albeit non-significant, association with job satisfaction (ß = 0.114, 95% CI = −0.080 to 0.308, *p* = 0.249). Chief doctors remained significantly associated with lower job satisfaction scores compared to assistant doctors (ß = −0.774, 95% CI = −1.536 to −0.012, *p* = 0.046), while working in a university clinic remained positively associated with job satisfaction (ß = 0.245, 95% CI = 0.001 to 0.490, *p* = 0.049). The effect of being in a partnership on job satisfaction lost significance in the multivariate analysis (ß = 0.152, 95% CI = −0.104 to 0.408, *p* = 0.244).

These findings suggest that working as a chief doctor was significantly associated with lower job satisfaction, while working in a university clinic was associated with higher job satisfaction. Other sociodemographic factors such as gender and age did not exhibit statistically significant associations with job satisfaction after adjustment for other factors.

## 4. Discussion

This study aimed to explore the job satisfaction, burnout, and well-being of first-generation migrant physicians in anesthesia and intensive care medicine in Germany.

Our findings indicate that job satisfaction levels of migrant and German physicians were largely similar across most dimensions measured by the German version of the Warr-Cook-Wall (WCW) Job Satisfaction Scale. Notably, there were no significant differences in satisfaction with physical workload, freedom to choose work methods, satisfaction with colleagues, responsibility, income, skill utilization, or the overall level of variety in work tasks. However, a significant difference was observed in satisfaction with the recognition received for work and working hours. Migrant physicians reported significantly higher satisfaction with the recognition they received compared to their German counterparts. Participants with a migration background had a mean satisfaction rating of 3.17 ± 1.80, whereas those without a migration background showed a higher dissatisfaction with a mean of 3.75 ± 1.68 (*p* < 0.001). This finding suggests that despite the challenges migrant physicians might face, they perceive the recognition they receive more positively compared to German physicians. Conversely, a significant difference was found in satisfaction with working hours, with migrant physicians reporting a mean satisfaction rating of 3.27 ± 1.79, while those without a migration background had a mean of 3.62 ± 1.73 (*p* = 0.035). This indicates that German physicians are more dissatisfied with their working hours compared to migrant physicians. This finding may reflect different expectations and work–life balance needs, suggesting that migrant physicians might have different coping mechanisms or perceptions regarding working hours.

The burnout ratings assessed using the Copenhagen Burnout Inventory (CBI) revealed significant differences in certain aspects of personal, work-related, and patient-related burnout between the two groups. While levels of tiredness, physical exhaustion, and emotional exhaustion were similar, migrant physicians reported significantly higher frequencies of thinking “I can’t take it anymore,” suggesting a higher psychological strain. Additionally, migrant physicians perceived every work hour as more exhausting and had significantly less energy for family and friends during their free time. These findings highlight the intense pressure migrant physicians may face, which can be exacerbated by additional stressors such as language barriers, cultural differences, and potential isolation from support networks [28]. These results align with a recent study that showed slightly higher burnout scores in employees with a migration background compared to those without a migration background in Germany [29]. Other stressors related to communication, cultural difficulties, and social integration may persist and impact burnout scores [30,31]. Additionally, physicians from other countries may also be exposed to workplace discrimination [32]. There are multiple potential stressors, and future research needs to explore which mechanisms are important for different groups of physicians in terms of cultural background, age, gender, etc., at various stages of the acculturation process. Some physician behaviors might serve as helpful resources; for example, a recent German study found that urologists with a migration background exhibited a lower risk of burnout when they engaged in reading non-medical books [33].

Moreover, migrant physicians reported significantly higher difficulty and frustration in working with patients, feeling more drained of energy, and wondering how much longer they could continue working with patients compared to their German counterparts. These aspects of patient-related burnout emphasize the need for targeted interventions to support migrant physicians in their interactions with patients, possibly through cultural competence training and peer support programs [34].

The well-being ratings assessed using the WHO Well-Being Index (WHO-5) showed some significant differences between the two groups. Migrant physicians reported lower levels of feeling energetic and active compared to their German counterparts, which may reflect the cumulative impact of stressors unique to their experiences. Interestingly, migrant physicians reported higher levels of having a daily life filled with things that interested them, suggesting that despite the challenges, they might find their work intrinsically rewarding or have developed effective coping mechanisms [35]. However, the overall lower ratings in feeling fresh and rested, as well as being happy and in good spirits, indicate an area of concern that requires attention to improve the overall well-being of migrant physicians. Lower satisfaction among migrant physicians in areas such as work atmosphere, relationships with colleagues, and social status has been noted in other authors [3]. Language barriers and cultural differences can hinder integration into the medical team and lead to misinterpretations of professional behavior, potentially resulting in conflicts and a perceived lack of competence. This phenomenon is not unique to Germany; international medical graduates in the USA also report similar experiences of workplace bias, discrimination, and limited professional opportunities [36,37].

The findings of this study contribute to a growing body of research on the experiences of migrant healthcare professionals in Germany. While few quantitative studies have focused specifically on first-generation migrant physicians in anesthesiology and intensive care, several semi-quantitative and qualitative surveys provide important context for understanding the broader experiences of migrant healthcare workers. For instance, Klingler’s [38] qualitative study on foreign-born and foreign-trained physicians highlighted significant challenges related to linguistic, cultural, and systemic knowledge, which often hindered their integration into the workplace. These findings align with our results, where migrant physicians reported greater psychological strain and frustration, particularly in patient-related burnout. Similarly, Klingler’s work underscores how the behavior of colleagues and superiors can exacerbate feelings of alienation and dissatisfaction—an issue that may be further compounded by inadequate recognition for migrant physicians, as indicated in our study. Roth et al. [39] extended these observations to internationally trained nurses, finding that many nurses migrated with the expectation of improved working conditions and professional development opportunities. However, unmet expectations often led to lower workplace satisfaction and hindered their integration. Our study mirrors these findings, particularly in terms of migrant physicians experiencing dissatisfaction with their working hours and overall well-being, which could similarly affect their integration and willingness to remain in Germany. These observations are consistent with broader European studies [38,40,41,42], which have examined the labor market integration of migrant healthcare professionals. Factors such as language acquisition, recognition of qualifications, and onboarding processes are critical for facilitating successful integration, and deficits in these areas can lead to dissatisfaction and burnout. While much of this research has focused on nurses, the challenges faced by migrant physicians in our study suggest that similar integration barriers exist across different healthcare roles, further emphasizing the need for targeted interventions.

The practical implications of our findings are significant. Measures aimed at improving job satisfaction and well-being among migrant physicians should be prioritized to retain qualified healthcare professionals and enhance patient care. Addressing burnout and recognition-related dissatisfaction could involve providing more structured support in the form of cultural competence training, clearer paths for professional development, and more flexible working conditions. Additionally, healthcare institutions must ensure that workplace policies are free from discriminatory practices and structural inequalities, as highlighted in studies by Can et al. [43]. Increasing awareness of the unique challenges faced by migrant healthcare professionals is crucial. For example, management-level interventions, such as promoting diversity and inclusivity in the workplace, may help to alleviate some of the psychological strain experienced by migrant physicians. Additionally, improving recognition for the contributions of migrant healthcare workers could help bridge gaps in job satisfaction, as emphasized by both our findings and the broader literature.

This study has several limitations that should be acknowledged. First, the cross-sectional design limits the ability to draw causal inferences from the observed associations. Second, the reliance on self-reported data may introduce response bias, as participants might provide socially desirable answers or misinterpret questions. However, the online format allowed respondents to complete the survey at their convenience, contributing to a higher response rate and reducing potential biases associated with time constraints. Third, the sample was drawn from a specific geographic area (Germany) and specialty, which may limit the generalizability of the findings to other regions or medical specialties. The study population included full-time employed migrant and German physicians to allow for comparative analysis. The actual working hours were not obtained through the survey, but only full-time employment respondents were considered. Given the strict working regulations in Germany, we assume that the actual workload of physicians was generally similar, although subjective perceptions of workload might differ. In terms of sample sufficiency, we conducted a post hoc power analysis to assess whether the sample size was adequate for detecting medium to large effects in the primary outcome variables (well-being, burnout, and job satisfaction). The power analysis indicated that the sample provided sufficient power (above 0.80), demonstrating that the sample size was adequate for the relationships investigated in the study. However, we acknowledge that the modest sample size may limit the detection of smaller, more nuanced effects. While the sample was sufficient to identify medium to large effects, it may have lacked the power to detect smaller associations that could be present between certain demographic or professional variables and the outcomes of interest. Additionally, the limited sample size may affect the generalizability of the findings, particularly when extrapolating to larger or more diverse populations of migrant physicians. Future research with larger, more diverse samples will be necessary to confirm the robustness of these findings and to explore potential smaller effects or interactions that were beyond the scope of the present analysis. Regarding response biases, while the study relied on voluntary participation, we compared the demographic characteristics of the sample with available national data on migrant physicians in Germany [43] to ensure that the participants were broadly representative of the target population. Despite the possibility of non-response bias, the distribution of responses across key demographic and professional variables, such as gender, years of experience, and migration background, suggests that our sample was representative of the population under investigation. This comparison helps to mitigate concerns regarding response bias and supports the generalizability of the results to similar populations. Finally, the study did not explore the potential impact of workplace policies or institutional support mechanisms on job satisfaction, burnout, and well-being, which could be significant factors in understanding the experiences of migrant physicians. Future research should consider these organizational factors to provide a more comprehensive understanding of the working conditions of migrant physicians.

The results of this study underscore the importance of recognizing and addressing the unique challenges faced by first-generation migrant physicians in the healthcare system. However, this study also highlights several avenues for future research that could further elucidate the complexities of migrant physician experiences and inform targeted interventions. First, future studies should investigate the longitudinal effects of burnout and job satisfaction among migrant physicians. While our cross-sectional design provided valuable insights into the current state of well-being and job satisfaction, longitudinal research could capture how these dynamics evolve over time and identify critical intervention points. Second, comparative studies between different medical specialties could provide a more nuanced understanding of the challenges specific to various fields of medicine. While this study focused on anesthesiology and intensive care medicine, other specialties may present distinct stressors or levels of job satisfaction that merit further exploration. Third, future research should explore the impact of institutional policies and support systems on mitigating burnout and enhancing job satisfaction. This includes examining the effectiveness of workplace interventions such as mentorship programs, cultural competence training, and flexible working arrangements across diverse healthcare settings. Additionally, more research is needed to examine the patient-care dynamics experienced by migrant physicians, particularly in relation to cultural competence and communication. Investigating how patient-related burnout can be mitigated through training programs that improve intercultural communication skills could help enhance the physician–patient relationship and overall job satisfaction. Finally, future studies should aim to capture the perspectives of a more diverse range of migrant physicians. Expanding the sample to include physicians from different countries of origin and regions within Germany could provide a deeper understanding of how regional healthcare practices and cultural differences shape the experiences of migrant physicians. By pursuing these objectives, future research can offer a more comprehensive understanding of how to improve the work environment and overall well-being of migrant physicians, ultimately benefiting healthcare systems and patient outcomes.

## 5. Conclusions

In conclusion, while first-generation migrant physicians in anesthesiology and intensive care medicine in Germany experience similar levels of job satisfaction to their German counterparts in many areas, they face distinct challenges in recognition, subjective workload, and burnout. Addressing these issues through targeted interventions can enhance the work experience and well-being of migrant physicians, ultimately contributing to better healthcare outcomes. Further research is needed to explore the long-term impact of these interventions and to identify additional strategies to support migrant physicians in the healthcare workforce.

## Figures and Tables

**Table 1:** Demographic characteristics and professional qualifications of participants with and without a migration background. The table includes mean age with standard deviation, gender distribution, place of residence, marital status, number of children, living arrangements, religious affiliation, additional professional qualifications, current job position, and type of clinic. Responses are presented as counts with corresponding percentages and mean ± standard deviation where applicable. Chi-squared and Mann–Whitney U tests were applied (non-normally distributed continuous variables).

Question	Response	Migrants	Germans	*p*-Value
How old are you?		36.5 ± 6.8	37.1 ± 9.0	0.492
Gender				
	Diverse/other	1 (0.9%)	1 (0.2%)	0.012
	Male	66 (60.0%)	213 (52.9%)	
	Female	41 (37.3%)	189 (46.9%)	
Where do you live?				
	Rural/small town	43 (39.1%)	167 (41.4%)	0.149
	Urban	66 (60.0%)	236 (58.6%)	
Marital status				
	Divorced	5 (4.5%)	4 (1.0%)	0.006
	In a partnership	19 (17.3%)	113 (28.0%)	
	Single	21 (19.1%)	90 (22.3%)	
	Married	64 (58.2%)	194 (48.1%)	
	Widowed	0 (0.0%)	2 (0.5%)	
How many children do you have?		1 ± 1	1 ± 1	0.322
Do your children live with you?				
	Yes	49 (86.0%)	157 (87.7%)	0.771
	No	8 (14.0%)	21 (11.7%)	
Which religion do you belong to?				
	Other	9 (8.2%)	1 (0.2%)	<0.001
	Christian	32 (29.1%)	219 (54.3%)	
	Jewish	1 (0.9%)	1 (0.2%)	
	None/agnostic/atheist	34 (30.9%)	177 (43.9%)	
	Muslim	33 (30.0%)	5 (1.2%)	
Emergency medicine qualification				
	Yes	43 (39.1%)	257 (63.8%)	<0.001
	No	67 (60.9%)	146 (36.2%)	
Intensive care medicine Qualification				
	Yes	90 (81.8%)	311 (77.2%)	0.296
	No	20 (18.2%)	92 (22.8%)	
Pain therapy qualification				
	Yes	104 (94.5%)	383 (95.0%)	0.835
	No	6 (5.5%)	20 (5.0%)	
Other qualification				
	Yes	95 (86.4%)	342 (84.9%)	0.695
	No	15 (13.6%)	61 (15.1%)	
No additional qualification				
	Yes	56 (50.9%)	278 (69.0%)	<0.001
	No	54 (49.1%)	125 (31.0%)	
Current position				
	Assistant doctor	54 (49.1%)	240 (59.6%)	0.048
	Chief doctor	1 (0.9%)	8 (2.0%)	
	Specialist doctor	34 (30.9%)	85 (21.1%)	
	Senior consultant	2 (1.8%)	16 (4.0%)	
	Consultant	18 (16.4%)	54 (13.4%)	
Type of clinic				
	Public clinic	79 (71.8%)	273 (67.7%)	0.134
	Private clinic	13 (11.8%)	43 (10.7%)	
	University clinic	17 (15.5%)	87 (21.6%)	

**Table 2:** Responses to the acculturation questionnaire items among participants with a migrant background, showing the distribution of language usage in various contexts. Responses are presented as counts with corresponding percentages in parentheses.

Question	Both Equally	More German than My Mother Tongue(s)	More My Mother Tongue(s) than German	German Only	Only My Mother Tongue(s)
What language(s) do you generally read and speak?	43 (39.1%)	24 (21.8%)	33 (30.0%)	8 (7.3%)	1 (0.9%)
Which languages did you use as a child?	7 (6.4%)	5 (4.5%)	10 (9.1%)	3 (2.7%)	84 (76.4%)
What language(s) do you speak at home?	16 (14.5%)	13 (11.8%)	26 (23.6%)	11 (10.0%)	43 (39.1%)
In which language(s) do you normally think?	33 (30.0%)	13 (11.8%)	24 (21.8%)	11 (10.0%)	28 (25.5%)
What language(s) do you normally speak with your friends?	30 (27.3%)	21 (19.1%)	30 (27.3%)	16 (14.5%)	12 (10.9%)
What language(s) are the TV programs you normally watch in?	30 (27.3%)	20 (18.2%)	26 (23.6%)	22 (20.0%)	11 (10.0%)
In which language(s) are the radio programs you normally listen to?	15 (13.6%)	20 (18.2%)	24 (21.8%)	44 (40.0%)	6 (5.5%)
In which language(s) are films, television, and radio programs broadcast that you prefer to watch?	27 (24.5%)	14 (12.7%)	30 (27.3%)	26 (23.6%)	11 (10.0%)

**Table 3:** Responses to social integration items of the acculturation questionnaire among participants with a migrant background, showing the distribution of social preferences and interactions. Responses are presented as counts with corresponding percentages in parentheses.

Question	All from My Home Country	Both Equally	More from My Home Country than from Germany	More German than from My Home Country	German Only
Close friends are:	21 (19.1%)	32 (29.1%)	28 (25.5%)	15 (13.6%)	11 (10.0%)
The persons who visit you or who are visited by you are:	19 (17.3%)	29 (26.4%)	27 (24.5%)	25 (22.7%)	9 (8.2%)
If you could choose your children’s friends, where would they come from?	4 (3.6%)	74 (67.3%)	16 (14.5%)	8 (7.3%)	4 (3.6%)
Prefer social gatherings/parties where people are from:	8 (7.3%)	58 (52.7%)	27 (24.5%)	14 (12.7%)	2 (1.8%)

**Table 4:** Job satisfaction ratings of participants with and without a migration background, based on the German version of the Warr-Cook-Wall (WCW) Job Satisfaction Scale. The table includes the mean and standard deviation of responses for each job satisfaction item. Participants rated their satisfaction on a Likert scale from 1 (very satisfied) to 7 (very dissatisfied). The items cover various aspects of job satisfaction including physical workload, freedom in work methods, satisfaction with colleagues, recognition received, responsibility, income, skill utilization, working hours, variety in work tasks, and overall job satisfaction. Comparisons were made using the Mann–Whitney U test for these Likert-scale items [27].

Question	Migrants	Germans	*p*-Value
How satisfied are you with the physical workload?	3.39 ± 1.70	3.13 ± 1.37	0.285
How satisfied are you with the freedom to choose your own work methods?	3.05 ± 1.58	3.10 ± 1.52	0.641
How satisfied are you with your colleagues and coworkers?	2.55 ± 1.41	2.40 ± 1.31	0.338
How satisfied are you with the recognition you receive for your work?	3.17 ± 1.80	3.75 ± 1.68	<0.001
How satisfied are you with the amount of responsibility you are given?	2.93 ± 1.47	3.05 ± 1.42	0.405
How satisfied are you with your income?	3.30 ± 1.83	3.25 ± 1.62	0.877
How satisfied are you with the opportunity to use your skills?	2.78 ± 1.41	2.77 ± 1.34	0.984
How satisfied are you with your working hours?	3.27 ± 1.79	3.62 ± 1.73	0.035
How satisfied are you with the level of variety in your work tasks?	2.94 ± 1.36	2.75 ± 1.41	0.094
Overall, how satisfied are you with your job?	3.00 ± 1.50	2.87 ± 1.35	0.551

**Table 5:** Burnout ratings of participants with and without a migration background, based on the Copenhagen Burnout Inventory (CBI). The table includes the mean and standard deviation of responses for each burnout item, categorized into personal, work-related, and patient-related burnout. Participants rated the frequency of their experiences on a Likert scale from 1 (almost never) to 7 (always). Comparisons were made using the Mann–Whitney U test for these Likert-scale items [27].

Question	Migrants Mean ± SD	Germans Mean ± SD	*p*-Value
Personal burnout			
How often do you feel tired?	3.67 ± 0.75	3.67 ± 0.70	0.964
How often are you physically exhausted?	3.37 ± 0.78	3.33 ± 0.80	0.612
How often are you emotionally exhausted?	3.08 ± 0.92	3.08 ± 0.90	0.882
How often do you think, “I can’t take it anymore”?	2.43 ± 1.08	2.18 ± 1.10	0.032
How often do you feel exhausted?	3.16 ± 0.89	3.24 ± 0.85	0.221
How often do you feel weak and susceptible to illness?	2.77 ± 0.98	2.54 ± 1.00	0.052
Work-related burnout			
Is your work emotionally demanding?	3.28 ± 0.88	3.32 ± 0.74	0.902
Do you feel burned out from your work?	2.73 ± 1.08	2.60 ± 0.97	0.285
Does your work frustrate you?	2.60 ± 0.95	2.69 ± 0.95	0.267
Do you feel exhausted at the end of your workday?	3.40 ± 0.90	3.49 ± 0.87	0.147
Are you exhausted in the morning at the thought of another workday?	2.77 ± 1.00	2.57 ± 1.08	0.089
Do you feel that every work hour is exhausting for you?	2.35 ± 0.91	2.03 ± 0.94	<0.001
Do you have enough energy for family and friends in your free time?	2.94 ± 0.91	3.20 ± 0.87	0.006
Patient-related burnout			
Is it difficult for you to work with patients?	1.96 ± 0.87	1.67 ± 0.71	0.001
Do you find it frustrating to work with patients?	1.93 ± 0.91	1.82 ± 0.81	0.402
Does working with patients drain your energy?	2.19 ± 0.99	2.00 ± 0.85	0.112
Do you feel that you give more than you get back when working with patients?	2.51 ± 1.05	2.56 ± 1.03	0.541
Are you tired of working with patients?	1.83 ± 0.91	1.52 ± 0.77	<0.001
Do you sometimes wonder how much longer you can work with patients?	1.98 ± 1.04	1.63 ± 0.87	<0.001

**Table 6:** Well-being ratings of participants with and without a migration background, based on the WHO Well-Being Index (WHO-5). The table includes the mean and standard deviation of responses for each well-being item. Participants rated their experiences on a Likert scale from 1 (all the time) to 6 (at no time). Comparisons were made using the Mann–Whitney U test for these Likert-scale items [27].

Question	Migrants Mean ± SD	Germans Mean ± SD	*p*-Value
In the past two weeks, I have been happy and in good spirits	2.80 ± 1.04	2.94 ± 1.06	0.245
In the past two weeks, I have felt calm and relaxed	3.19 ± 1.26	3.41 ± 1.16	0.075
In the past two weeks, I have felt energetic and active	3.24 ± 1.25	3.58 ± 1.18	0.008
In the past two weeks, I have felt fresh and rested when waking up	3.72 ± 1.32	3.94 ± 1.32	0.097
In the past two weeks, my daily life has been filled with things that interest me	3.43 ± 1.24	3.11 ± 1.21	0.017

**Table 7:** Descriptive statistics for WHO-5 well-being, Copenhagen Burnout Inventory (CBI), and Warr-Cook-Wall (WCW) Job Satisfaction scales. This table presents the Spearman’s Rho correlation coefficients between the overall scale scores of WHO-5, CBI, and WCW. SD = standard deviation; CI = confidence interval; IQR = interquartile range.

Variables	Mean ± SD	95% CI of the Mean	Median	Min	Max	IQR
WHO-5 Well-Being Total Score	18.14 ± 4.84	[17.72, 18.56]	19.00	6.00	29.00	7.00
Normalized CBI Score (0–100 scale)	40.17 ± 13.26	[39.02, 41.33]	38.16	7.89	82.89	17.11
Mean Job Satisfaction Score (WCW)	3.06 ± 1.06	[2.97, 3.16]	2.90	1.00	7.00	1.55

**Table 8:** Correlation matrix for WHO-5 well-being, Copenhagen Burnout Inventory (CBI), and Warr-Cook-Wall (WCW) Job Satisfaction scales. This table presents the Spearman’s Rho correlation coefficients between the overall scale scores of WHO-5, CBI, and WCW. ** *p* < 0.01.

Variables	WHO-5 Well-Being Total Score	Normalized CBI Score (0–100 Scale)	Mean Job Satisfaction Score (WCW)
WHO-5 Well-Being Overall Scale Score	1.000	−0.689 **	−0.559 **
CBI Overall Scale Score	−0.689 **	1.000	0.586 **
Job Satisfaction (WCW) Overall Scale Score	−0.559 **	0.586 **	1.000

**Table 9:** Univariate and multivariate linear regression analysis of WHO-5 well-being overall scale score. This table presents the results of both univariate and multivariate linear regression analyses, showing the association between various independent variables (age, gender, migration background, marital status, number of children, religious affiliation, qualifications, current position, and type of clinic) and the WHO-5 well-being overall scale score. ß represents the regression coefficient, and *p*-values indicate the statistical significance of the associations. The reference categories for the categorical variables are noted in the table. The 95% confidence intervals (CI) for the multivariate model are also provided.

	WHO-5 Well-Being Overall Scale Score				
	Univariate Linear Regression Analysis		Multivariate Linear Regression Analysis		
Variables	ß	*p* Value	ß	95% CI	*p* Value
How old are you?	0.056	0.024	0.022	−0.039 to 0.082	0.488
Gender (male versus female)	−1.424	<0.001	−1.476	−2.353 to −0.599	0.130
Migration background (yes versus no)	−0.616	0.242	0.936	−0.275 to 2.146	0.700
Where do you live? (rural/small town versus urban)	−1.109	0.011	−0.872	−1.750 to 0.006	0.052
Marital status	-	-	-	-	-
Married	Reference				
Divorced	−0.777	<0.001	−1.483	−4.862 to 1.897	0.389
In a partnership	−0.501	0.333	0.175	−0.966 to 1.317	0.763
Single	−0.523	0.345	0.596	−0.660 to 1.853	0.352
Widowed	4.098	0.232	4.674	−2.031 to 11.379	0.171
How many children do you have?	0.454	0.012	0.206	−0.290 to 0.701	0.415
Do your children live with you? (yes versus no)	−0.169	0.698	-	-	-
Which religion do you belong to?	-	-	-	-	-
None/agnostic/atheist	Reference				
Christian	0.874	0.022	0.685	−0.208 to 1.578	0.132
Jewish	0.538	0.086	5.064	−1.596 to 11.725	0.136
Muslim	−0.162	0.848	−1.430	−3.348 to 0.487	0.143
Other	17.662	<0.001	3.388	−0.120 to 6.897	0.058
Emergency medicine qualification (no versus yes)	0.476	0.274	-	-	-
Intensive care medicine qualification (no versus yes)	0.058	0.912	-	-	-
Pain therapy qualification (no versus yes)	0.922	0.342	-	-	-
Other qualification (no versus yes)	−0.240	0.693	-	-	-
No additional qualification (no versus yes)	−0.123	0.784	-	-	-
Current position	-	-	-	-	-
Assistant doctor	Reference				
Chief doctor	1.997	0.221	-	-	-
Specialist doctor	0.142	0.788	-	-	-
Senior consultant	1.719	0.143	-	-	-
Consultant	0.222	0.728	-	-	-
Type of clinic	-	-	-	-	-
Public clinic	Reference				
Private clinic	−0.182	0.807	-	-	-
University clinic	−0.303	0.576	-	-	-

**Table 10:** Univariate and multivariate linear regression analysis of Copenhagen Burnout Inventory (CBI) Scores. This table presents the results of both univariate and multivariate linear regression analyses, showing the associations between various independent variables (age, gender, migration background, marital status, number of children, religious affiliation, qualifications, current position, and type of clinic) and the Copenhagen Burnout Inventory (CBI) score. The table provides the regression coefficient (ß), *p*-values, and 95% confidence intervals (CI) for the multivariate analysis. The reference categories for the categorical variables are specified in the table.

	Copenhagen Burnout Inventory (CBI)				
	Univariate Linear Regression Analysis		Multivariate Linear Regression Analysis		
Variables	ß	*p* Value	ß	95% CI	*p* Value
How old are you?	−0.123	0.074	0.006	−0.158 to 0.169	0.947
Gender (male versus female)	4.805	<0.001	4.377	2.002 to 6.752	<0.001
Migration background (yes versus no)	2.089	0.150	2.601	−0.214 to 5.416	0.070
Where do you live? (rural/small town versus urban)	0.257	0.831	-	-	-
Marital status	-	-	-	-	-
Married	Reference				
Divorced	4.235	0.374	-	-	-
In a partnership	2.132	0.136	-	-	-
Single	2.389	0.117	-	-	-
Widowed	−10.732	0.254	-	-	-
How many children do you have?	−1.339	0.007	−0.988	−2.186 to 0.211	0.106
Do your children live with you? (yes versus no)	−1.386	0.249	-	-	-
Which religion do you belong to?	-	-	-	-	-
None/agnostic/atheist	Reference				
Christian	−0.720	0.563	-	-	-
Jewish	−11.931	0.205	-	-	-
Muslim	3.983	0.093	-	-	-
Other	−0.089	0.985	-	-	-
Emergency medicine qualification (no versus yes)	−0.910	0.448	-	-	-
Intensive care medicine qualification (no versus yes)	−1.041	0.467	-	-	-
Pain therapy qualification (no versus yes)	−0.189	0.944	-	-	-
Other qualification (no versus yes)	−0.098	0.954	-	-	-
No additional qualification (no versus yes)	−0.055	0.965	-	-	-
Current position	-	-	-	-	-
Assistant doctor	Reference				
Chief doctor	−5.691	0.206	-	-	-
Specialist doctor	0.270	0.853	-	-	-
Senior consultant	−2.913	0.853	-	-	-
Consultant	1.446	0.411	-	-	-
Type of clinic	-	-	-	-	-
Public clinic	Reference				
Private clinic	−0.815	0.673	-	-	-
University clinic	−0.098	0.948	-	-	-

**Table 11:** Univariate and multivariate linear regression analysis of Warr-Cook-Wall (WCW) Job Satisfaction scores. This table presents the results of both univariate and multivariate linear regression analyses, showing the associations between various independent variables (age, gender, migration background, marital status, number of children, religious affiliation, qualifications, current position, and type of clinic) and the Warr-Cook-Wall (WCW) Job Satisfaction score. The table provides the regression coefficient (ß), *p*-values, and 95% confidence intervals (CI) for the multivariate analysis. The reference categories for the categorical variables are specified in the table.

	Warr-Cook-Wall (WCW) Job Satisfaction Scales				
	Univariate Linear Regression Analysis		Multivariate Linear Regression Analysis		
Variables	ß	*p* Value	ß	95% CI	*p* Value
How old are you?	−0.008	0.132	0.006	−0.011 to 0.022	0.498
Gender (male versus female)	0.178	0.058	0.114	−0.080 to 0.308	0.249
Migration background (yes versus no)	−0.058	0.615	−0.037	−0.267 to 0.193	0.752
Where do you live? (rural/small town versus urban)	0.159	0.096	0.083	−0.114 to 0.281	0.408
Marital status	-	-	-	-	-
Married	Reference				
Divorced	0.126	0.740	0.101	−0.653 to 0.856	0.792
In a partnership	0.225	0.047	0.152	−0.104 to 0.408	0.244
Single	0.108	0.371	0.019	−0.260 to 0.299	0.892
Widowed	0.026	0.972	−0.198	−1.694 to 1.298	0.795
How many children do you have?	−0.076	0.056	−0.015	−0.125 to 0.094	0.786
Do your children live with you? (yes versus no)	−0.027	0.775	-	-	-
Which religion do you belong to?	-	-	-	-	-
None/agnostic/atheist	Reference				
Christian	−0.145	0.143	-	-	-
Jewish	−0.582	0.439	-	-	-
Muslim	0.031	0.867	-	-	-
Other	−0.182	0.633	-	-	-
Emergency medicine qualification (no versus yes)	−0.091	0.337	-	-	-
Intensive care medicine qualification (no versus yes)	−0.006	0.959	-	-	-
Pain therapy qualification (no versus yes)	−0.255	0.230	-	-	-
Other qualification (no versus yes)	0.049	0.710	-	-	-
No additional qualification (no versus yes)	0.024	0.810	-	-	-
Current position	-	-	-	-	-
Assistant doctor	Reference				
Chief doctor	−0.834	0.019	−0.774	−1.536 to −0.012	0.046
Specialist doctor	0.074	0.519	0.124	−0.138 to 0.385	0.354
Senior consultant	−0.601	0.019	−0.486	−1.070 to 0.099	0.103
Consultant	−0.174	0.209	−0.104	−0.440 to 0.232	0.544
Type of clinic	-	-	-	-	-
Public clinic	Reference				
Private clinic	0.102	0.503	0.094	−0.214 to 0.403	0.548
University clinic	0.303	0.010	0.245	0.001 to 0.490	0.049

## Data Availability

The data presented in this study are available upon request from the corresponding author.

## Supplementary Materials

The following supporting information can be downloaded at: https://www.mdpi.com/article/10.3390/healthcare12212107/s1, Table S1: Mean ± Standard Deviation and *p*-values for WHO-5, CBI, and WCW Scores by Independent Variables. The *p*-values represent the results of pairwise comparisons between the categories. Statistically significant *p*-values (*p* < 0.05) are indicated for the respective comparisons.; Table S2: Spearman’s Correlation Matrix for Age and Number of Children with WHO-5 Well-Being, CBI, and WCW Scores. This table shows the Spearman’s correlation coefficients between Age, Number of Children, and the three dependent variables (WHO-5 Well-Being Total Score, CBI Score, and Job Satisfaction Score). Significant correlations at the *p* < 0.05 level are indicated by *, while correlations significant at the *p* < 0.01 level are indicated by **.
