# Supplementary material for: Job Satisfaction Among First-Generation Migrant Physicians in Anesthesiology and Intensive Care Medicine in Germany
Source: Healthcare (Basel). 2024 Oct 23;12(21):2107. doi: 10.3390/healthcare12212107 (PMC11545438; doi:10.3390/healthcare12212107)
Supplement: Supplementary file 1 [file healthcare-12-02107-s001.zip › healthcare-3224178-supplementary.pdf]

Supplementary Table S1: Mean ± Standard Deviation and p-values for WHO-5, CBI, and WCW Scores by Independent Variables. The p-values represent the results of pairwise comparisons between the categories. Statistically significant p-values ($p < 0.05$) are indicated for the respective comparisons.

Dependent Variable	Independent Variable	Category	Mean±Std	p-value
WHO5_Total	Migration background	Yes	18.62 ± 5.07	0.154
		No	18.01 ± 4.77	
	Gender	Diverse/Other	17.0 ± 9.9	0.004
		Male	18.81 ± 4.71	
		Female	17.38 ± 4.83	
	Where do you live?	Rural/Small Town	18.84 ± 4.77	0.003
		Urban	17.66 ± 4.83	
	Marital Status	Divorced	18.33 ± 6.38	0.403
		In a partnership	17.9 ± 4.44	
		Single	17.74 ± 5.27	
		Married	18.4 ± 4.79	
		Widowed	22.5 ± 0.71	
	Do your children live with you?	Yes	18.22 ± 4.84	0.27
		No	18.06 ± 4.84	
	Which religion do you belong to?	Other	20.7 ± 4.08	0.021
		Christian	18.5 ± 4.91	
		Jewish	23.5 ± 0.71	
		None/Agnostic/Atheist	17.66 ± 4.67	
		Muslim	17.5 ± 5.15	
	Emergency Medicine Qualification	No	17.85 ± 4.82	0.179
		Yes	18.35 ± 4.84	
	Intensive Care Medicine Qualification	No	18.15 ± 4.73	0.754
		Yes	18.14 ± 5.21	
	Pain Therapy Qualification	No	18.1 ± 4.81	0,228
		Yes	19.04 ± 5.28	
	Other Qualification	No	18.21 ± 4.71	0.714
		Yes	17.75 ± 5.52	
	No Additional Qualification	No	18.18 ± 4.94	0.588
		Yes	18.08 ± 4.64	
CBI_Normalized	Migration background	Yes	41.98 ± 14.24	0.219
		No	39.68 ± 12.96	
	Gender	Diverse/Other	53.95 ± 1.86	<0.001
		Male	37.94 ± 13.3	
		Female	42.75 ± 12.77	
	Where do you live?	Rural/Small Town	40.03 ± 14.02	0.783
		Urban	40.27 ± 12.72	

	Marital Status	Divorced	44.59 ± 21.06	0.156
		In a partnership	41.15 ± 12.13	
		Single	41.54 ± 14.04	
		Married	39.02 ± 13.1	
		Widowed	28.29 ± 0.93	
	Do your children live with you? Which religion do you belong to?	Yes	39.29 ± 13.22	0.081
		No	40.9 ± 13.21	
	Which religion do you belong to?	Other	42.89 ± 10.36	0.139
		Christian	39.52 ± 13.05	
		Jewish	28.29 ± 8.37	
		None/Agnostic/Atheist	40.22 ± 13.58	
		Muslim	44.2 ± 13.23	
	Emergency Medicine Qualification	No	40.66 ± 13.23	0.682
		Yes	39.82 ± 13.29	
	Intensive Care Medicine Qualification	No	40.38 ± 12.87	0.579
		Yes	39.41 ± 14.59	
	Pain Therapy Qualification	No	40.18 ± 13.18	0.723
		Yes	39.93 ± 14.89	
	Other Qualification	No	40.16 ± 12.87	0.939
		Yes	40.25 ± 15.35	
	No Additional Qualification	No	40.23 ± 13.57	0.68
		Yes	40.07 ± 12.7	
WCW_Mean	Migration background	Yes	3.04 ± 1.16	0.725
		No	3.07 ± 1.03	
	Gender	Diverse/Other	4.3 ± 0.42	0.032
		Male	2.98 ± 1.07	
		Female	3.16 ± 1.04	
	Where do you live?	Rural/Small Town	2.97 ± 1.02	0.109
		Urban	3.13 ± 1.08	
	Marital Status	Divorced	3.2 ± 1.24	0.332
		In a partnership	3.2 ± 1.0	
		Single	3.1 ± 1.11	
		Married	2.97 ± 1.05	
		Widowed	3.0 ± 0.0	
	Do your children live with you?	Yes	3.04 ± 1.02	0.539
		No	3.09 ± 1.08	

	Which religion do you belong to?	Other	3.22 ± 1.13	0.290
		Christian	2.99 ± 1.06	
		Jewish	2.55 ± 0.49	
		None/Agnostic/Atheist	3.13 ± 1.03	
		Muslim	3.16 ± 1.22	
	Emergency Medicine Qualification	No	3.11 ± 1.09	0.34
		Yes	3.03 ± 1.04	
	Intensive Care Medicine Qualification	No	3.06 ± 1.03	0.614
		Yes	3.07 ± 1.14	
	Pain Therapy Qualification	No	3.08 ± 1.06	0.15
		Yes	2.82 ± 0.98	
	Other Qualification	No	3.05 ± 1.01	0.937
		Yes	3.13 ± 1.32	
	No Additional Qualification	No	3.06 ± 1.08	0.682
		Yes	3.07 ± 1.01	

*Supplementary Table S2: Spearman's Correlation Matrix for Age and Number of Children with WHO-5 Well-Being, CBI, and WCW Scores. This table shows the Spearman's correlation coefficients between Age, Number of Children, and the three dependent variables (WHO-5 Well-Being Total Score, CBI Score, and Job Satisfaction Score). Significant correlations at the $p < 0.05$ level are indicated by *, while correlations significant at the $p < 0.01$ level are indicated by **.*

Variables	Age	Number of Children
WHO-5 Well-Being Total Score	0.059	0.097*
Normalized CBI Score (0-100 scale)	-0.019	-0.102*
Mean Job Satisfaction Score (WCW)	-0.023	-0.071
Age	1.000	0.634**
Number of Children	0.634**	1.000
